# The NF-*κ*B pathway: regulation of the instability of atherosclerotic plaques activated by Fg, Fb, and FDPs

**DOI:** 10.1007/s11010-013-1751-2

**Published:** 2013-07-10

**Authors:** Yongjun Cao, Xiaomei Zhou, Huihui Liu, Yanlin Zhang, Xiaoyan Yu, Chunfeng Liu

**Affiliations:** 1 Department of Neurology, The Second Affiliated Hospital of Soochow University, No. 1055, Sanxiang Road, Suzhou, 215004 Jiangsu People’s Republic of China; 2 Institute of Neuroscience, Soochow University, Suzhou, 215123 People’s Republic of China

**Keywords:** Fibrin(ogen), Fibrinogen degradation products, Atherosclerosis, Matrix metalloproteinase, Vascular endothelial growth factor, Nuclear factor-kappa B

## Abstract

Recently, the molecular mechanism responsible for the instability of atherosclerotic plaques has gradually become a hot topic among researchers and clinicians. Matrix metalloproteinases (MMPs) and vascular endothelial growth factor (VEGF) play an important role in the processes of formation and development of atherosclerosis. In this study, we established and employed the transwell co-culture system of rabbit aortic endothelial cells and smooth muscle cells to explore the relationship between fibrin (Fb), fibrinogen (Fg), and/or their degradation products (FDPs) in relation to the instability of atherosclerotic plaques; meanwhile, we observed the effects of Fg, Fb, and FDPs on the mRNA levels of MMPs and VEGF as well as on the activation of nuclear factor-kappa B (NF-*κ*B). We concluded that Fb, Fg, and FDPs are involved in the progression of the instability of atherosclerotic plaques via increasing the expression of MMPs and VEGF. This effect might be mediated by the NF-*к*B pathway.

## Introduction

The instability of atherosclerotic plaques is an important factor leading to the rupture of plaques and the occurrence of stroke, and has been drawing increasing attention of researchers and clinicians. Epidemiological studies have demonstrated that hyperfibrinogenemia is an independent risk factor for stroke, as well as that fibrinogen (Fg), fibrin (Fb), and fibrinogen degradation products (FDPs) are involved in the formation and development of atherosclerosis (AS) [1, 2]. Fg is a 340 kD glycoprotein which is constitutively expressed exclusively in hepatocytes [3]. As a key plasma protein, Fg has a plasma half-life of 3–5 days and can be converted to Fb in the final step of the coagulation cascade [4]. FDPs are fragments (polypeptides) produced when either Fg or Fb is broken down by the enzyme plasmin [5].

Numerous studies have demonstrated that the plasma levels of Fg, Fb, and FDPs have close association with vascular diseases. People with high levels of Fg are more than twice as likely to die of a heart attack or stroke as people with normal Fg levels [6]. Wada et al. [7] found that the plasma level of Fb monomer in patients with disseminated intravascular coagulation (DIC) was significantly higher than that in patients with pre-DIC or in non-DIC patients. Gaffney et al. [8] reported that the levels of FDPs can rise after any thrombotic event, indicating it can be used to test for DIC. Despite the findings of these studies, however, the relationship between Fg, Fb, and FDPs and the instability of atherosclerotic plaques remains unclear. The aim of the current study was to explore the molecular mechanisms of Fg, Fb, and FDPs as they are involved in the instability of atherosclerotic plaques. We established and employed the transwell co-culture system of rabbit aortic endothelial cells (ECs) and smooth muscle cells (SMCs) to investigate the relationship between Fg, Fb, and/or their degradation products (FDPs) in relation to the instability of atherosclerotic plaques.

## Materials and methods

### Materials

Healthy male rabbits (2 months old, weighing 2–3 kg) were purchased from the Animal Center of Soochow University (Suzhou, China). Rabbit SMCs were purchased from the Institute of Basic Medical Sciences of Chinese Academy of Medical Sciences, China. Dulbecco’s modified eagle medium (DMEM) was purchased from Gibco Company, USA. Fetal bovine serum (FBS) was purchased from Hyclone Company, USA. Endothelial cell growth supplement (ECGS), Type II collagenase, and parenzyme were purchased from Sigma Company, USA. 3-(4,5)-Dimethylthiahiazo(-z-y1)-3,5-di-phenytetrazoliumromide (MTT) was purchased from Fluka Company, Sweden. NF-*κ*B inhibitor SN50 was purchased from UBI Company, USA. RiboPure™ minimal RNA extraction kit was purchased from Ambion Company, USA. Agents for reverse transcription and polymerase chain reaction (PCR) were purchased from Shanghai Sangon Biological Engineering Technology and Service Co. Ltd, China. Mouse polyclonal antibody to human VIII factor and mouse monoclonal anti-rabbit *α*-SM-actin was purchased from Lab Vision Company, USA. Relevant secondary antibodies were purchased from Wuhan Boster Biological Engineering Co. Ltd, China. VEGF, MMP-2, and MMP-8 enzyme-linked immunosorbent assay (ELISA) kits were purchased from BPB Biology Company, USA. NF-*κ*B active-nucleus transit detection kit was purchased from Nantong Biyuntian Biological Technology Co. Ltd, China. Transwell 0.4 and 8 μm-pore size gelatinized polycarbonate membranes were purchased from Corning Company, USA.

### Cell isolation and culture

Primary aortic endothelial cells were isolated from the thoracic aortae of healthy male rabbits according to a previously described method [9]. ECs between the 3rd and 5th passage and SMCs between the 7th and 10th passage were used for the experiments. Both ECs and SMCs were cultured in a humidified 95 % air, 5 % CO_2_ incubator at 37 °C and maintained in DMEM supplemented with 10 % FBS.

### Experimental groups and preparation of Fg, Fb, and FDPs

The solutions of Fg, Fb, and FDPs at various concentrations were prepared using a previously described method [10]. The cultured cells were divided into three experimental groups: Fg, Fb, and FDPs intervention groups. Each group was subdivided into 6 subgroups (0, 0.5, 1.5, 3.0, 4.5, and 6.0 mg/ml) according to the concentrations of Fg, Fb, and FDPs. SN50 (50 μg/ml) was administered to the groups, in which NF-*κ*B was activated, 1 h before adding Fg, Fb, and FDPs.

### Establishment of the rabbit aorta ECs–SMCs co-culture system

Rabbit aorta ECs–SMCs co-culture system was established according to our previous study [11]. ECs (10 × 10^4^ cells/ml) in logarithmic periods were seeded onto Transwell inserts, and then put into 12-well plates; SMCs (10 × 10^4^ cells/ml) were cultured into the bottom chamber of 12-well plates and then cultured to confluence. After being cultured in serum-free DMEM for 24 h, the Transwell membranes cultured with confluent ECs were inserted into plates cultured with SMCs, thus, the ECs–SMCs co-culture system was established. ECs were seeded into the inner chamber with 300 μl DMEM, and SMCs in the outer chamber with 1200 μl, they were then incubated with Fg, Fb, or FDPs (outer chamber) for 24 h. SN50 was introduced into the chambers 1 h before adding Fg, Fb, or FDPs. After treatment, the supernatant was gathered and stored at −70 °C. Total mRNA was extracted from the co-cultured cells according to the one-step method (Trizol agent).

### NF-*κ*B activation

Inactive NF-*κ*B is a cytoplasmic complex that binds with κB inhibitor (I*κ*B)-*α*. Once activated, I*κ*B-*α* is phosphated and degradated by the ubiquitin–proteasome pathway. The separation of I*κ*B-*α* from NF-*κ*B exposes the sequence binding the nucleus, which leads to the translocation of NF-*κ*B into the nucleus and the transcription of NF-*κ*B-dependent genes. The activation of NF-*κ*B is detected by the ability of its main subunit, p65, to translocate from cytoplasm into nuclei using immunofluorescence staining. Here, NF-*κ*B p65 is stained with red fluorescence and the nucleus was stained with blue.

To establish the ECs–SMCs indirect co-culture system, SMCs were seeded onto the coverslips (18 mm × 18 mm) in the bottom of 6-well plates, and ECs were seeded onto 0.4 mm pore size Transwell inserts. The confluent cells were cultured with serum-free DMEM for 24 h, then treated with Fg, Fb, or FDPs (0.5, 3.0, or 6.0 mg/ml) for 24 h. After treatment, the cells were fixed with 4 % paraformaldehyde, packed in silver papers, and stored at −20 °C for staining.

Cells were rinsed in 0.1 M phosphate buffered saline (PBS) at room temperature (RT) and then incubated in blocking buffer for 1 h. Subsequently, the section was incubated with anti-NF-*κ*B-p65 antibody at 4 °C overnight, followed by anti-rabbit Cy3 at RT for 1 h. Later, the section was incubated with 2-(4-Amidinophenyl)-6-indolecarbamidine dihydrochloride (DAPI) at RT for 5 min before mounting. NF-*κ*B was stained red and the nucleus was stained with DAPI blue. The merger of red and blue suggests that NF-*κ*B p65 has transferred into the nucleus and has been activated.

### Examination of MMP-2 mRNA and VEGF mRNA

Total RNA was extracted from co-cultured cells strictly following the procedures outlined in the manual included in the kit. Reverse transcription was carried out in a 20 μl reactive system. PCR was performed with Taq DNA polymerase (30 cycles, 94 °C for 30 s, 55.5 °C for 30 s, and 72 °C for 30 s), followed by a final extension at 72 °C for 7 min in a 50 μl reactive system. Rabbit MMP-2 upstream primer: 5′-AGCCTTCTCACCCCCACCTG-3′, downstream primer: 5′-GCCCTTATCCCACTGCCCC-3′ [12]; Rabbit VEGF upstream primer: 5′-GACATCTTCCAGGAGTACCC-3′, downstream primer: 5′-TGAGGTTTGATCCGCATGAT-3′ [13]; GAPDH upstream primer: 5′-ACGAATTTGGCTACAGCAACAGG-3′, downstream primer: 5′-GGTCTGGGATGGAAACTGTGAAG-3′ [14]. The amplified fragments had an expected length of 313 bp, 157 bp, and 196 bp, respectively. The PCR reaction products (6 μl) were subjected to 1.5 % agarose gel electrophoresis and stained with ethidium bromide. The intensity of the specific bands was quantified by image analysis.

### Detection of contents of MMP-2, MMP-8, and VEGF in medium

The MMP-2, -8, and VEGF content in the medium were detected by cellular ELISA kits using a DG3022A-type cellular enzyme-linked immunosorbent detection apparatus at 450 nm. The standard curve was made according to the absorbance of standard samples, and the MMP-2, -8, and VEGF content was calculated using the standard curve.

### MMP-2 and VEGF activation assay

Levels and degrees of activation of MMP-2 were examined in co-cultured cells by use of the gelatin zymography method [15] on sodium dodecyl sulfate–polyacrylamide gel electrophoresis (SDS–PAGE) containing gelatin. Supernatant aliquots containing 150 μg of total protein were used. The mixture (25 μl) of supernatant aliquots and buffer solutions (1:1) was loaded onto a polyacrylamide gel (5 % stacking gel, 10 % separating gel containing 0.5 % gelatin). After electrophoresis, the gels were washed with 2.5 % TritonX-100 solution twice, 45 min each time, and then incubated with medium (containing 50 mmol/L Tris–HCl, 50 mmol/L NaCl, 10 mmol/L CaCl_2_, 1 μmol/L ZnCl_2_, 1 % TritonX-100, pH 7.6) at 37 °C for 48 h. The gels were stained with 0.05 % Coomassie Blue R-250 for 3 h before being decolored. MMP-2 band intensity was quantified by the Quantity One Image analysis system, and the data were expressed as the bands’ digestibility: bands digestibility = area of bands × (gray value of bands–gray value of background).

The activity of VEGF was determined by use of MTT methods [16]. ECs (1 × 10^5^ cells/ml) from the 3rd to 5th passages were pre-incubated with the gathered medium and standard VEGF for 48 h. Subsequently, MTT was added into the medium at a final concentration of 0.5 mg/ml, and the cells were incubated for another 4 h in a CO_2_ incubator at 37 °C. Resultant insoluble formazan crystal was dissolved in dimethyl sulfoxide (DMSO) at 37 °C for 30 min. With blank control at the zero-setting, the absorbance was detected at 570 nm (OD_570_). The activity of VEGF was expressed as OD_570_ value of samples/OD_570_ value of standards.

### Statistical analysis

For all quantitative experiments, statistical analyses of data were performed using either an unpaired t test or a one-way analysis of variance (ANOVA) by SPSS11.5 software. Values are cited as mean ± standard deviation (SD). *P* < 0.05 is considered a statistically significant probability.

## Results

### NF-*κ*B activation

**Fig. 1:**
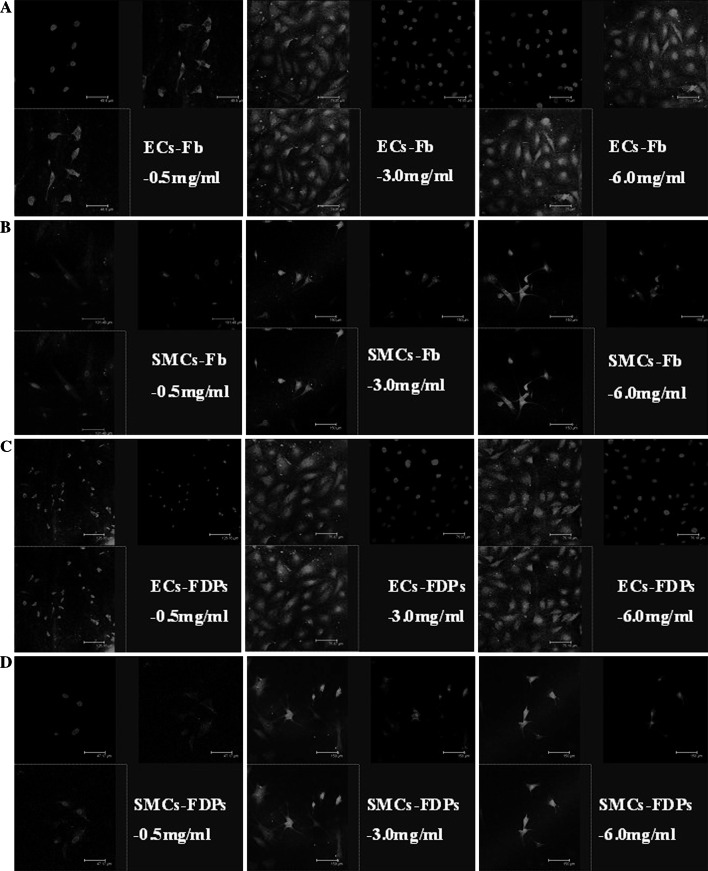
The effect of Fg, Fb, and FDPs on the activation of NF-*κ*B in ECs and SMCs. ECs between the 3rd and 5th passage and SMCs between the 7th and 10th passage were used for the experiments. Both ECs and SMCs were cultured in DMEM containing 10 % FBS. Red fluorescence indicates NF-*κ*B p65 staining and blue indicates DAPI staining. **a** 3.0 and 6.0 mg/ml, but not 0.5 mg/ml of Fb initiated the activation of NF-*κ*B in ECs; **b** 3.0 and 6.0 mg/ml, but not 0.5 mg/ml of Fb initiated the activation of NF-*κ*B in SMCs; **c** 3.0 and 6.0 mg/ml, but not 0.5 mg/ml of FDPs initiated the activation of NF-*κ*B in ECs; **d** 3.0 and 6.0 mg/ml, but not 0.5 mg/ml of FDPs initiated the activation of NF-*κ*B in SMCs. Bar = 150 μm

Fb and FDPs (3.0–6 mg/ml) activated NF-*κ*B of ECs and SMCs. However, the activated NF-*κ*B was not found in the Fg groups, as well as the Fb and FDPs groups whose concentrations were lower than 0.5 mg/ml. (Fig. 1).

### mRNA levels of MMP-2 and VEGF

**Fig. 2:**
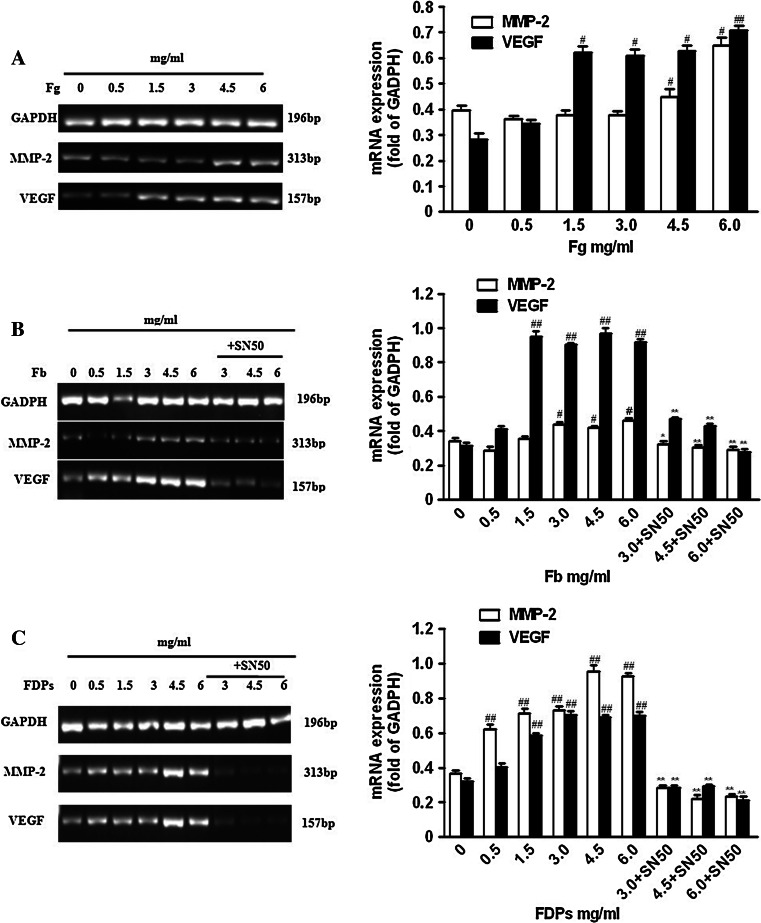
The effects of Fg, Fb, and FDPs on the mRNA levels of MMP-2 and VEGF in co-cultured ECs–SMCs. The establishment of ECs–SMCs co-culture system was described in the “Materials and methods” section. **a** Fg could significantly increase the mRNA levels of MMP-2 and VEGF in co-cultured ECs–SMCs. **b** Fb could significantly increase the mRNA levels of MMP-2 and VEGF in co-cultured ECs–SMCs, and the treatment of 50 μg/ml SN50, NF-κB inhibitor, showed inhibitory effect against this increase. **c** FDPs could significantly increase the mRNA levels of MMP-2 and VEGF in co-cultured ECs–SMCs, and the treatment of 50 μg/ml SN50, NF-*κ*B inhibitor, showed inhibitory effect against this increase. Data are shown as mean ± SD for six replicates (*n* = 6), which are a representative of three independent experiments. ^#^ *P* < 0.05 and ^##^ *P* < 0.01, compared with the groups without Fg, Fb, or FDPs treatment; ^*^ *P* < 0.05 and ^**^ *P* < 0.01, compared with the groups without SN50 treatment

The mRNA level of MMP-2 was upregulated with an increase on culture concentrations when exposed to Fg (4.5–6.0 mg/ml), Fb (3.0–6.0 mg/ml), and FDPs (0.5–6.0 mg/ml). VEGF mRNA level had a similar effect under treatments of Fg, Fb (1.5–6.0 mg/ml), and FDPs (0.5–6.0 mg/ml). NF-*κ*B inhibitor SN50 treatment prevented the upregulation of their mRNA levels (Fig. 2).

### MMP-2, MMP-8, and VEGF contents

**Fig. 3:**
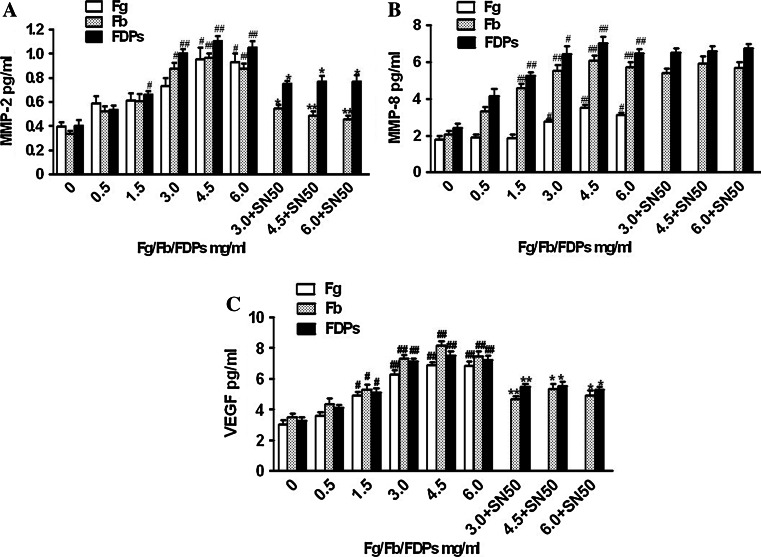
MMP-2, MMP-8, and VEGF contents in the supernatant of medium in which ECs–SMCs were co-cultured. The contents were assayed by ELISA kit. The establishment of ECs–SMCs co-culture system was described in the “Materials and methods” section. **a** Treatment with Fg, Fb, and FDPs at various concentrations could induce a significant increase in the content of MMP-2 in the supernatant of medium, and the treatment of 50 μg/ml SN50, NF-κB inhibitor, showed inhibitory effect against this increase induced by Fb and FDPs. **b** Treatment with Fg, Fb, and FDPs at various concentrations could induce a significant increase in the content of MMP-8 in the supernatant of medium, but the treatment of 50 μg/ml SN50, NF-*κ*B inhibitor, did not have inhibitory effect against this increase induced by Fb and FDPs. **c** Treatment with Fg, Fb, and FDPs at various concentrations could induce a significant increase in the content of VEGF in the supernatant of medium, and the treatment of 50 μg/ml SN50, NF-*κ*B inhibitor, showed inhibitory effect against this increase induced by Fb and FDPs. Data are shown as mean ± SD for five replicates (*n* = 5), which are a representative of three independent experiments. ^#^ *P* < 0.05 and ^##^ *P* < 0.01, compared with the groups without Fg, Fb, or FDPs treatment; ^*^ *P* < 0.05 and ^**^ *P* < 0.01, compared with the groups without SN50 treatment

MMP-2, MMP-8, and VEGF contents were nearly increased as a result of an increase in culture concentrations in the (4.5–6.0), (3.0–4.5), and (1.5–6.0) mg/ml Fg groups; only MMP-8 was slightly decreased in the 6.0 mg/ml Fg group; MMP-2 content increased significantly in the 3.0–6.0 mg/ml Fb groups, as well as MMP-8 and VEGF contents in the 1.5–6.0 mg/ml Fb groups; FDPs resulted in a markedly increased content of MMP-2, -8, and VEGF that began at 1.5 mg/ml and reached its peak at 4.5 mg/ml, and then declined after 6.0 mg/ml. SN50 decreased the contents of MMP-2 and VEGF in the Fb and FDPs (4.5–6.0 mg/ml) groups, but no changes in MMP-8 levels were detected in SN50-treated groups (Fig. 3).

### Activity of MMP-2 and VEGF

**Fig. 4:**
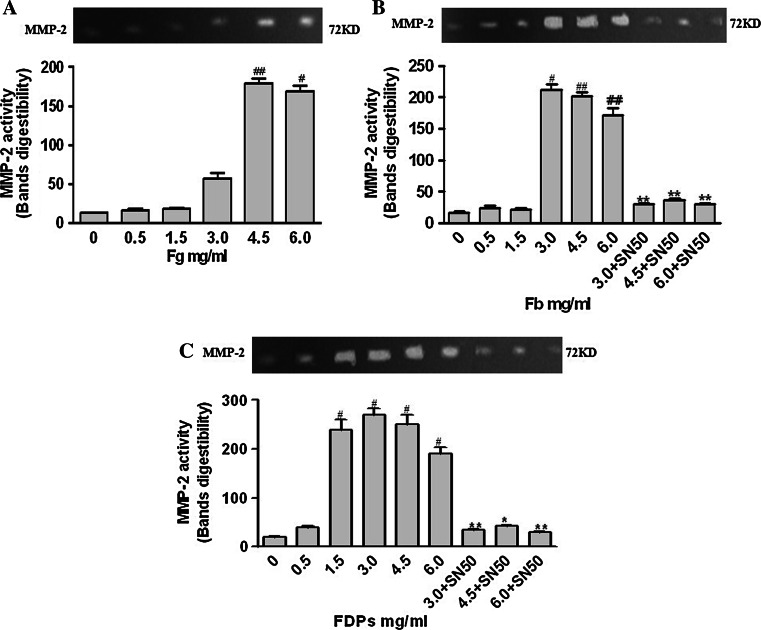
Activity of MMP-2 and VEGF in the supernatant of medium in which ECs–SMCs were co-cultured. The activity was assayed by gelatin zymography method. Supernatant aliquots containing 150 μg of total protein were used. The establishment of ECs–SMCs co-culture system was described in the “Materials and methods” section. **a** Fg enhanced the activity of MMP-2 in the supernatant. **b** Fb enhanced the activity of MMP-2 in the supernatant, and the treatment of 50 μg/ml SN50, NF-*κ*B inhibitor, showed inhibitory effect against this enhancement. **c** FDPs enhanced the activity of MMP-2 in the supernatant, and the treatment of 50 μg/ml SN50, NF-*κ*B inhibitor, showed inhibitory effect against this enhancement. Data are presented as mean ± SD for four replicates (*n* = 4), which are a representative of three independent experiments. Bands digestibility = area of bands × (gray value of bands–gray value of background). ^#^ *P* < 0.05 and ^##^ *P* < 0.01, compared with the groups without Fg, Fb, or FDPs treatment; ^*^ *P* < 0.05 and ^**^ *P* < 0.01, compared with the groups without SN50 treatment

**Fig. 5:**
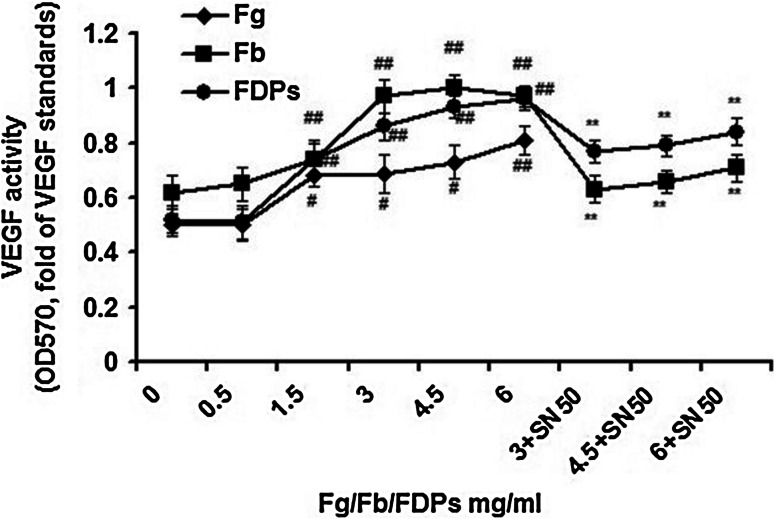
Activity of VEGF in the supernatant of ECs medium. The activity was assayed by MTT methods. ECs (1 × 10^5^ cells/ml) from the 3rd to 5th passages were used for the experiment. Fg enhanced the activity of VEGF in the supernatant; Fb/FDPs enhanced the activity of VEGF in the supernatant, and the treatment of 50 μg/ml SN50, NF-*κ*B inhibitor, showed inhibitory effect against this enhancement. Data are presented as mean ± SEM for eleven replicates (*n* = 11), which are a representative of three independent experiments. The activity of VEGF was expressed as OD_570_ value of samples/OD_570_ value of standards. ^#^ *P* < 0.05 and ^##^ *P* < 0.01, compared with the groups without Fg, Fb, or FDPs treatment; ^**^ *P* < 0.01, compared with the groups without SN50 treatment

Fb (4.5–6.0 mg/ml) and FDPs (1.5–6.0 mg/ml) enhanced the activity of MMP-2 and VEGF. The activity of MMP-2 increased with 4.5–6.0 mg/ml Fg and VEGF increased with 1.5–6.0 mg/ml. SN50 inhibited the effect of Fb and FDPs (3.0–6.0 mg/ml) (Figs. 4, 5).

## Discussion

At present, the molecular mechanisms responsible for the instability of atherosclerotic plaques have become a hot topic among clinicians and researchers. Instability of atherosclerotic plaques implies two meanings: (a) the shaded plaques are prone to cause congestion in small vascular compartments; (b) the instability of plaques is apt to form thrombosis, which results in further occlusion of vessels. The degradation of fibrous caps by MMPs and neovascularization in plaques are major events involving in the instability of atherosclerotic plaques. Multiple studies have demonstrated that MMP-2, MMP-8, and VEGF play an important role in the instability of atherosclerotic plaques [17, 18].

Fibrinogen is one of the independent reconcilable factors leading to stroke. An increase in plasma Fg and its degradation products, Fb and FDPs, can stimulate the proliferation and transformation of SMCs, leading to the sclerosis of vessel walls and the narrowing of vessels. Elevated Fg, Fb, and FDPs in fibrous caps also contribute to the rupture of atherosclerotic plaques by enhancing endothelial permeability and migration of SMCs [2]. Along these lines, we find that the instability of plaques is related to the mRNA levels and contents of MMPs and VEGF associated with the degradation of matrices and neovascularization. Thus, we hypothesize that the injury of ECs and the proliferation and migration of SMCs are accompanied by the degradation of matrices and neovascularization [19].

Matrix metalloproteinases-2 (MMP-2) is recognized as an important degradation enzyme of type IV glutin protein. Moreover, active MMP-2 contributes to the neovascularization through its degradation of matrices [20]. In the progression of AS, MMP-2 is involved in the migration of vascular SMCs from the media to the intima through the basement membrane, which mainly consists of type IV glutin protein. Recent studies demonstrate that there is increased expression and activity of MMP-2 in human instable atherosclerotic plaques [21]. Therefore, MMP-2 plays an important role in the instability of atherosclerotic plaques through regulating the degradation of plaque matrices and neovascularization. In our study, we used the vascular ECs–SMCs co-culture system and found that high levels (4.5–6.0 mg/ml) of Fg dramatically increased the mRNA level, content, and activity of MMP-2. Consistent with this finding, Fb (3.0–6.0 mg/ml) obviously enhanced the mRNA level, content, and activity of MMP-2. As far as FDPs (0.5–6.0 mg/ml) were concerned, the mRNA level of MMP-2 was upregulated in a concentration-dependent manner. However, the increase in content and activity of MMP-2 was found only in the 1.5–6.0 mg/ml FDPs groups, suggesting there may exist post-transcriptional regulatory factors that can regulate the mRNA-protein-activity chain of MMP-2.

Matrix metalloproteinases-8 (MMP-8) is recognized as a degradated enzyme and mainly degradates type I–VI glutin protein, elastin, fibronectin, and laminin. Its ability to degradate glutin protein is twice that of other interstitial collagenases [22]. Growing evidence indicates that MMP-8 is expressed in the ECs and SMCs of human atherosclerotic plaques, cooperates with macrophage cells leading to the degradation of type I glutin, and coexists with their degradation fragments in the “shoulder” of plaques. These results suggest that MMP-8 plays an important role in the instability of atherosclerotic plaques [23–25]. Furthermore, MMP-8 not only prompts the degradation of matrix via activating MMP-2, -3, and -9 [26], but also regulates the expression of cytokine factors IL-1β, IL-8, TNF-α, and T cell membrane proteins CD2, 4, and 8, thereby aggravating the inflammatory response [27]. In our present work, we showed that the level of MMP-8 increased in a concentration-dependent manner when exposed to Fg (3.0–6.0 mg/ml) Fb, and FDPs (1.5–6.0 mg/ml), and reached a peak at 4.5 mg/ml. However, the mRNA level and activity of MMP-8 were not examined because of the absence of its rabbit primer and activity detection method.

Neovascularization has been recognized as a cardinal feature of unstable plaques. So far, VEGF is the only growth factor tested that has a specific role in cellular karyokinesis, and its effect in the neovascularization has been attentioned by increasingly more scholars. The effects of VEGF in neovascularization may be due to its ability to promote the expression of MMPs (mainly MMP-2), degradate the basic vascular membrane, and enhance the migration of ECs [28]. In the early stages, VEGF can improve the impairment of vascular intima by stimulating neovascular formation in atherosclerotic plaques [29]. However, with the increasing neovascular ingrowth in plaques, all kinds of inflammation factors and lipoprotein deposits are aggregated, which further lead to the degradation of matrices, thinning of fibrous caps, and cause plaques to become brittle and rupture. Therefore, the role played by VEGF in clinical diseases is due to the balance it exerts on improvement and proliferation of VECs, which is the balance between atherosclerotic plaques’ progress and neovascular formation. Consistent with this, we found that Fg (>1.5 mg/ml), Fb, and FDPs all enhanced the mRNA level and content of VEGF as well as its subsequent activity.

Inflammation plays a central role in the progress of AS. NF-κB induces the expression of many inflammatory mediators as a necessary transcription factor [30]. Related expression productions induced by NF-*κ*B contribute to the formation and development of AS at the site of inflammation by aggregating vascular inflammation and causing it to linger. The inflammation pathway induced by NF-*κ*B family is a common method by which many risk factors promote the development of AS [31]. In our study, we found that only Fb (3.0–6.0 mg/ml) and FDPs (3.0–6.0 mg/ml) resulted in the enhanced activation of NF-*κ*B of ECs and SMCs, but Fg and lower levels of Fb and FDPs did not. We next explored the relationship between the activation of NF-*κ*B and the mRNA levels and contents of MMP-2, -8, and VEGF using the NF-*κ*B inhibitor SN50 and found that SN50 could decrease the mRNA levels and contents of MMP-2 and VEGF, but not MMP-8. Therefore, we concluded that Fb and FDPs regulate the expression of MMP-2 and VEGF via an NF-*κ*B-dependent pathway.

However, we are pressed to ask why SN50 does not affect the mRNA level and content of MMP-8, and why Fb and FDPs could activate the NF-*κ*B pathway in ECs and SMCs, but not Fg? There are two small peptides, Aα_1–16_ and Bβ_1–14_, which have been designated fibrinopeptides A and B (FPA, FPB) in the N-terminal regions of FgAα and Bβ chains. The interaction of thrombin with Fb causes the release of the two small peptides, which results in the formation of Fb. Aα_1–16_ and Bβ_1–14_ are the only differences between Fg and Fb [32]. However, neither the cleavage of these peptides that changes the crystal structure of Fg or Fb, nor the one which activates the NF-*κ*B pathway are not clear. More recently, mitogen-activated protein (MAP) kinases and NF-*κ*B have been identified as important molecules that regulate the transduction of the Fgγ_117–133_/ICAM-1 signal pathway. Previous studies also report that protein kinase C (PKC), the upstream of MAPK, is involved in the regulation of the VE-cadherin/FbBβ_15–42_ signal pathway [33]. These results suggest that Fg, Fb, and FDPs may regulate the mRNA levels, contents, and subsequent activities of MMP-2 and VEGF through the PKC and MAPK-mediated activation of NF-*к*B.

In this study, we show that Fg, Fb, and FDPs are involved in the progress of the instability of atherosclerotic plaques by increasing the mRNA levels, contents, and activities of MMP-2, -8, and VEGF. This effect may be mediated by the NF-*к*B pathway. Regulation of the expression of these proteins and the NF-*к*B pathway may have a favorable effect on the treatment and prognosis of AS.

## References

[1] Fukujima MM, Martinez TL, Pinto LE, Auriemo Cdo R, de Andrade LA (1997). Fibrinogen as independent risk factor for ischemic stroke. Arq Neuropsiquiatr.

[2] Mauriello A, Sangiorgi G, Palmieri G, Virmani R, Holmes DR, Schwartz RS, Pistolese R, Lppoliti A, Spagnoli LG (2000). Hyperfibrinogenemia is associated with specific histocytological composition and complications of atherosclerotic carotid plaques in patients affected by transient ischemic attacks. Circulation.

[3] Reinhart WH (2003). Fibrinogen—marker or mediator of vascular disease?. Vasc Med.

[4] Levenson J, Giral P, Megnien JL, Gariepy J, Plainfosse MC, Simon A (1997). Fibrinogen and its relations to subclinical extracoronary and coronary atherosclerosis in hypercholesterolemic men. Arterioscler Thromb Vasc Biol.

[5] Koenig W (1999). Fibrinogen and coronary risk. Curr Cardiol Rep.

[6] Packard CJ, O’Reilly DS, Caslake MJ, McMahon AD, Ford I, Cooney J, Macphee CH, Suckling KE, Krishna M, Wilkinson FE, Rumley A, Lowe GD (2000). Lipoprotein-associated phospholipase A2 as an independent predictor of coronary heart disease. West of Scotland Coronary Prevention Study Group. N Engl J Med.

[7] Wada H, Wakita Y, Nakase T, Shimura M, Hiyoyama K, Nagaya S, Deguchi H, Mori Y, Kaneko T, Deguchi K, Fujii J, Shiku H (1996). Increased plasma-soluble fibrin monomer levels in patients with disseminated intravascular coagulation. Am J Hematol.

[8] Gaffney PJ, Edgell T, Creighton-Kempsford LJ, Wheeler S, Tarelli E (1995). Fibrin degradation product (FnDP) assays: analysis of standardization issues and target antigens in plasma. Br J Haematol.

[9] Tashiro K, Shimokama T, Haraoka S, Tokunaga O, Watanabe T (1994). Endothelial cell heterogeneity in experimentally-induced rabbit atherosclerosis. Demonstration of multinucleated giant endothelial cells by scanning electron microscopy and cell culture. Virchows Arch.

[10] Cao YJ, Wu YH, Liu CF (2008). Effect of fibrinogen, fibrin and fibrin (ogen) degradation products on the tissue plasminogen activator and plasminogen activator inhibitor-1 expressions of vascular endothelial cells in coculture system. Zhonghua Xin Xue Guan Bing Za Zhi.

[11] Cao YJ, Qian JJ, Liu CF, Zhang ZL, Huo HM (2007). Establishment of the coculture systems of rabbit aortic endothelial cells and smooth muscle cells. Zhongguo Ying Yong Sheng Li Xue Za Zhi.

[12] Chen HF, Pu XD, Lin YZ, Lin LF, Wu ZY (2004). Dynamic changes of MMP-2, TIMP-2 mRNA after balloon injure in aortic arteries of rabbits. J Clin Cardiol (China).

[13] Zhu Y, Liu ZL (2003). A comparison of the mRNA expression of VEGF and its receptor FLK-1 in rabbit iris pigment epithelium cells and retinal pigment epithelial cells. Zhonghua Yan Ke Za Zhi.

[14] Inoue K, Cynshi O, Kawabe Y, Nakamura M, Miyauchi K, Kimura T, Daida H, Hamakubo T, Yamaguchi H, Kodama T (2002). Effect of BO-653 and probucol on c-MYC and PDGF-A messenger RNA of the iliac artery after balloon denudation in cholesterol-fed rabbits. Atherosclerosis.

[15] Amorino GP, Hoover RL (1998). Interactions of monocytic cells with human endothelial cells stimulate monocytic metalloproteinase production. Am J Pathol.

[16] Jiang B, Zhang YL, Zhou DY (1996). Basic methods in molecular biology.

[17] Cao YJ, Liu CF (2005). Vascular endothelial growth factor, matrix metalloproteinase-2, -8 and the instability of atherosclerotic plaques. Foreign Med Sci Cerebrovasc Dis.

[18] Cao YJ, Qian JJ, Liu CF (2008). The localization coexpression of matrix metalloproteinase-2, -8 and vascular endothelial growth factor in human unstable carotid atherosclerosis plaques. Chin J Neurol.

[19] Lorenzet R, Sobel JH, Bini A, Witte LD (1992). Low molecular weight fibrinogen degradation products stimulate the release of growth factors from endothelial cells. Thromb Haemost.

[20] Itoh T, Tanioka M, Yoshida H, Yoshioka T, Nishimoto H, Itohara S (1998). Reduced angiogenesis and tumor progression in gelatinase A-deficient mice. Cancer Res.

[21] Li Z, Li L, Zielke HR, Cheng L, Xiao R, Crow MT, Stetler-Stevenson WG, Froehlich J, Lakatta EG (1996). Increased expression of 72-kd type IV collagenase (MMP-2) in human aortic atherosclerotic lesions. Am J Pathol.

[22] Glass CK, Witztum JL (2001). Atherosclerosis: the road ahead. Cell.

[23] Herman MP, Sukhova GK, Libby P, Gerdes N, Tang N, Horton DB, Kilbride M, Breitbart RE, Chun M, Schonbeck U (2001). Expression of neutrophil collagenase (matrix metalloproteinase-8) in human atheroma: a novel collagenolytic pathway suggested by transcriptional profiling. Circulation.

[24] Molloy KJ, Thompson MM, Jones JL, Schwalbe EC, Bell PR, Naylor AR, Loftus IM (2004). Unstable carotid plaques exhibit raised matrix metalloproteinase-8 activity. Circulation.

[25] Men BZ, Zhou DB, Shi HY, Zhang XM (2004). The expression of MMP-8 in the atherosclerotic carotid plaques. Chin J Neurosci.

[26] Sukhova GK, Schonbeck U, Rabkin E, Schoen FJ, Poole AR, Billinghurst RC, Libby P (1999). Evidence for increased collagenolysis by interstitial collagenases-1 and -3 in vulnerable human atheromatous plaques. Circulation.

[27] Ferry G, Lonchampt M, Pennel L, de Nanteuil G, Canet E, Tucker GC (1997). Activation of MMP-9 by neutrophil elastase in an in vivo model of acute lung injury. FEBS Lett.

[28] Risau W (1997). Mechanisms of angiogenesis. Nature.

[29] Rosengart TK, Lee LY, Patel SR, Sanborn TA, Parikh M, Bergman GW, Hachamovitch R, Szulc M, Kligfield PD, Okin PM, Hahn RT, Devereux RB, Post MR, Hackett NR, Foster T, Grasso TM, Lesser ML, Isom OW, Crystal RG (1999). Angiogenesis gene therapy: phase I assessment of direct intramyocardial administration of an adenovirus vector expressing VEGF121 cDNA to individuals with clinically significant severe coronary artery disease. Circulation.

[30] Hilliard B, Samoilova EB, Liu TS, Rostami A, Chen Y (1999). Experimental autoimmune encephalomyelitis in NF-kappa B-deficient mice:roles of NF-kappa B in the activation and differentiation of autoreactive T cells. J Immunol.

[31] Collins T (1993). Endothelial nuclear factor-kappa B and the initiation of the atherosclerotic lesion. Lab Invest.

[32] Cao YJ, Qian JJ, Liu CF (2006). Fibrin(ogen), its degradation products and their motif antagonist and atherosclerosis. Int J Cerebrovasc Dis.

[33] Tsakadze NL, Zhao Z, D’Souza SE (2002). Interactions of intercellular adhesion molecule-1 with fibrinogen. Trends Cardiovasc Med.

